# CryoCycle your grids: Plunge vitrifying and reusing clipped grids to advance cryoEM democratization

**DOI:** 10.21203/rs.3.rs-4415026/v1

**Published:** 2024-07-02

**Authors:** Viacheslav Serbynovskyi, Jing Wang, Eugene YD Chua, Aygul Ishemgulova, Lambertus M. Alink, William C. Budell, Jake D. Johnston, Charlie Dubbeldam, Fabio A. Gonzalez, Sharon Rozovsky, Edward T. Eng, Alex de Marco, Alex J. Noble

**Affiliations:** 1 Simons Electron Microscopy Center, New York Structural Biology Center, NY, NY, USA; 2 Department of Cellular and Molecular Physiology & Biophysics, Columbia University, New York, NY, USA; 3 Department of Chemistry and Biochemistry, University of Delaware, Newark, DE, USA; 4 Biochemistry and Molecular Biophysics, Columbia University, New York, NY, USA

## Abstract

CryoEM democratization is hampered by access to costly plunge-freezing supplies. We introduce methods, called CryoCycle, for reliably blotting, vitrifying, and reusing clipped cryoEM grids. We demonstrate that vitreous ice may be produced by plunging clipped grids with purified proteins into liquid ethane and that clipped grids may be reused several times for different protein samples. Furthermore, we demonstrate the vitrification of thin areas of cells prepared on gold-coated, pre-clipped grids.

Cryo-electron microscopy (cryoEM) of biological specimens requires sample vitrification prior to imaging to preserve high-resolution details of biomolecular structures (Taylor & Glaeser, 1974; Frank, 2006). Conventional cryoEM grid preparation entails applying ~3 μL of protein sample to an unclipped EM grid, blotting away excess sample, and plunging the grid into liquid nitrogen (LN2)-cooled liquid ethane (commonly using commercial devices such as the Thermo Fisher Scientific (TFS) Vitrobot or Leica EM GP) (Dubochet & McDowall, 1981; Dobro et al., 2010). During the screening step to optimize conditions for high-resolution structure determination, grid and sample conditions are varied, dozens of grids are prepared, and grids are either screened in a side-loading cryo-transmission EM (cryoTEM) or clipped and screened in a cryoTEM with an autoloader (Sawh-Gopal et al., 2023). Each new screening condition requires one or more new grids and possibly autogrid rings and c-clips. However, initial and recurring grid preparation costs, along with availability limitations of consumables, restrict the process to sufficiently-funded institutions.

One solution to decrease grid preparation costs would be to reuse grid-autogrid assemblies (herein called ‘clipped grids’ in general or ‘pre-clipped grids’ if clipping occurs before freezing). We focused on developing methods to reuse clipped grids without disassembling them. The primary challenges with reusing clipped grids are 1) sample vitrification and 2) cleaning clipped grids. Regarding challenge (1), researchers in academia and industry have attempted to blot and vitrify pre-clipped grids consistently for over a decade without success and with minimal reporting in the literature (Ravelli et al., 2020). Regarding challenge (2), while a reported method exists for cleaning unclipped EM grids that also removes carbon film (Goldie et al., 2014), no method has been reported for cleaning unclipped or clipped EM grids that does not remove the film. This research gap stems from the absence of an affordable method for vitrifying clipped cryoEM grids, with the only available option being cost-prohibitive ethane jet vitrification, such as the implementation in the VitroJet (Ravelli et al., 2020).

We examined existing blotting methods to address challenge (1) by testing multiple blotting materials in multiple instruments (TFS Vitrobot Mark IV and Leica EM GP2) and found that no combination sufficiently thinned the ice to <100 nm uniformly nor consistently vitrified the ice (Supplementary Fig. 1). In these tests, a minority of grid holes contained thin, vitreous ice, and many contained broken and/or poorly hydrated ice. The former issue has been speculated to be due to the autogrid’s heat capacity being too high, thus impeding vitrification (Ravelli et al., 2020). For the latter issue, we suspect that the blotting paper fails to uniformly contact the grid, causing locally dry areas (Supplementary Fig. 1). This is due to the autogrid extending tens to hundreds of microns above the grid (Supplementary Fig. 2), causing blotting paper larger than the clipped grid to not uniformly contact the grid.

Here we demonstrate a reliable method for producing consistently thin, vitreous ice by applying sample to a clipped grid, blotting only the grid, and immediately freezing in liquid ethane (solution to challenge (1)) (Supplementary Video 1). Blotting the grid just inside of the autogrid assembly leaves a thin film of sample which may be vitrified by conventional plunge freezing. We introduce a modified pipette tip called a ‘blotting pipette tip’ to blot the grid inside of the clip ring (Fig. 1a,b; Supplementary Fig. 3). Subsequently, we demonstrate a method for reusing clipped grids multiple times by washing sequentially with water then isopropanol while shaking (solution to challenge (2)) (Fig. 2). We provide protocols for preparing (Supplementary Protocol 1) and reusing (Supplementary Protocol 2) clipped grids for single particle cryoEM. Furthermore, we show that the blotting method is sufficient for vitrifying thin areas of cells grown on grids and provide a protocol for preparing cell-compatible, pre-clipped grids (Supplementary Protocol 3). Figure 1c–h shows examples of vitrified samples of single particles (globular proteins and a long complex) and cells (human cells grown on grids; Supplementary Fig. 4) prepared on multiple different grid types using blotting pipette tips. We compared orientations of the p97/selenos complex prepared with a blotting pipette tip (Fig. 1f) versus conventionally (Supplementary Fig. 5a) and found them to be comparable (Supplementary Fig. 5b,c). These methods, called CryoCycle, significantly reduce costs and demand for consumables, thus democratizing cryoEM by enabling more widespread and uninterrupted adoption.

The key components for blotting clipped grids for vitrification are rigid, flat-tipped tweezers and a blotting pipette tip that uniformly contacts the grid. For the former, we found that conventional fine-tipped Vitrobot tweezers insecurely hold clipped grids due to their flexible tips, causing rotation into the tweezers when handled (Supplementary Fig. 6a,b). Trimming the tweezer tips increases rigidity sufficiently to handle the autogrid without rotating into the grid, which is essential for blotting (Supplementary Fig. 6a,c & 7; Supplementary Video 1; Supplementary Protocol 1). For the latter, a blotting pipette tip is created by cutting the narrow end of a pipette tip to a 3 mm inner diameter then inserting a ~1 cm piece of filter paper until 1 mm protrudes out of the small end (Fig. 1a,b; Supplementary Protocol 1). Rounding the protruding filter paper’s edges facilitates blotting within the clip ring, while flattening the end helps ensure uniform grid contact (Supplementary Fig. 8). 200 μL and 1,000 μL blotting pipette tips accommodate enough blotting paper to absorb 3+ μL of sample (Fig. 1a,b). Supplementary Video 2 shows the CryoCycle blotting pipette tip assembly process. Supplementary Figure 3 depicts sample application, blotting direction, and orientation of the grid and autogrid for single particle and cell samples.

We developed a washing protocol for reusing single particle clipped grids, taking into account the sensitivity of the grid film and the autogrid assembly. Clipped EM grids are typically composed of copper, carbon, gold, and palladium. Washing them first with water removes bulk contamination, then subsequent isopropanol washes displace and rinse away remaining samples and contaminants. Isopropanol was selected for its minimal reactivity with these metals at room temperature and high purity (99+%) to prevent altering their chemical composition. We determined that one 5-minute wash with water followed by two 5-minute isopropanol washes sufficiently cleans clipped carbon and gold grids (Fig. 2; Supplementary Fig. 9; Supplementary Protocol 2). Figure 2e shows a micrograph from a clipped carbon grid that was reused twice (i.e. 3 samples, 2 washings) and Supplementary Figure 10 shows their grid atlases with the vast majority of squares intact, exemplifying the protocol’s robustness. The maximum number of times clipped grids may be reused has not yet been determined.

The CryoCycle clipped grid preparation methods offer significant advantages over conventional unclipped grid preparation in four key areas. 1) *Room temperature grid clipping is simpler and safer*, as it avoids mechanical and visual disruptions caused by LN2, enables easy visual verification of clipped ring placement by eye, and eliminates risks of grids thawing, fingers freezing, and ice contamination. Additionally, a clean, flat surface can be used to clip instead of a clipping station, saving a one-time cost of thousands of dollars. 2) *Handling pre-clipped grids mitigates mechanical damage and user stress* (Supplementary Fig. 11) compared to handling unclipped grids (Supplementary Fig. 7); ideally, a grid is handled once for clipping, then only the autogrid is touched thereafter. Handling clipped grids substantially reduces bent grids, which expedites screening due to minimal defocus gradients. Moreover, clipped grid handling eliminates the need to purchase unclipped grid boxes. 3) *CryoCycle blotting and freezing only requires a humidity chamber*; popular commercial semi-automated plunge freezing devices are not needed, which may reduce one-time costs by tens of thousands of dollars. While we illustrate the use of CryoCycle in a Vitrobot, we emphasize that only the humidity chamber and plunger are used (Supplementary Video 1); CryoCycle grid preparation using a gravity plunger (Comolli et al., 2012; Depelteau et al., 2020) or manually plunging by hand is possible, although the latter has been minimally tested (Supplementary Fig. 12). 4) *Reusing clipped grids reduces initial and recurring costs*. Each pre-clipped grid assembly costs about $45 in 2024, so for a typical cryoEM sample where 24 grids are required to be screened before conditions suitable for data collection are found, reusing 8 grids three times each would reduce recurring costs by $720. Most cryoEM projects require optimization of several samples, thus using CryoCycle methods reduces costs of cryoEM projects by thousands of dollars per project and reduces costs for cryoEM labs by tens of thousands of dollars per year. Table 1 lists where CryoCycle methods can reduce both initial and yearly costs by tens of thousands of dollars each for typical cryoEM labs. Additionally, CryoCycle methods enable vitrification of clipped grids by plunge freezing, circumventing the previous requirement of using ethane jet freezers that cost hundreds of thousands of dollars. The advantages listed here particularly benefit new cryoEM users, making cryoEM more accessible.

## METHODS

### Sample Preparation

#### Virus-like Particles (VLPs):

PP7 Leviviridae PP7 VLPs were prepared as described in (Zhao et al., 2019) at ~1 mg/mL.

#### Apoferritin:

Mouse apoferritin stock (8 mg/mL) was provided by Dr. Kikkawa’s lab (U Tokyo).

#### Protein mixture:

Purified apoferritin and tobacco mosaic virus (TMV) were mixed in phosphate buffered saline at concentrations of 2.9 mg/mL and 13.6 mg/mL, respectively.

#### p97/selenos complex:

The AAA ATPase p97 was combined with an excess molar quantity of full-length selenoprotein S U188C (selenos), resulting in a final concentration of the p97/selenos complex at 11 mg/mL. This mixture was incubated on ice for 30 minutes, including 5 mM ATP*γ*S and 1.2 mM DDM (1 CMC), prior to grid application. The preparation of selenos followed the method outlined in (Ghelichkhani et al., 2023).

#### Human retinal pigment epithelial-1 (RPE-1) cells:

RPE-1 cells expressing IRFP::hCentrin2, mCherry::α-tubulin, sfGFP::CENP-A were acquired from the Needleman Lab (Harvard University) and cultured in DMEM (Thermo Fisher Scientific) supplemented with 10% FBS (Thermo Fisher Scientific) and Penicillin-Streptomycin (Thermo Fisher Scientific) in a humidified incubator with 5% CO_2_ maintained at 37°C. Cells were trypsinized and plated onto gold-coated, pre-clipped carbon Quantifoil grids (Supplementary Protocol 3) at a density of 1,000 cells per grid and grown overnight prior to freezing.

### Grid Preparation and Vitrification

**Figure 1 |:**
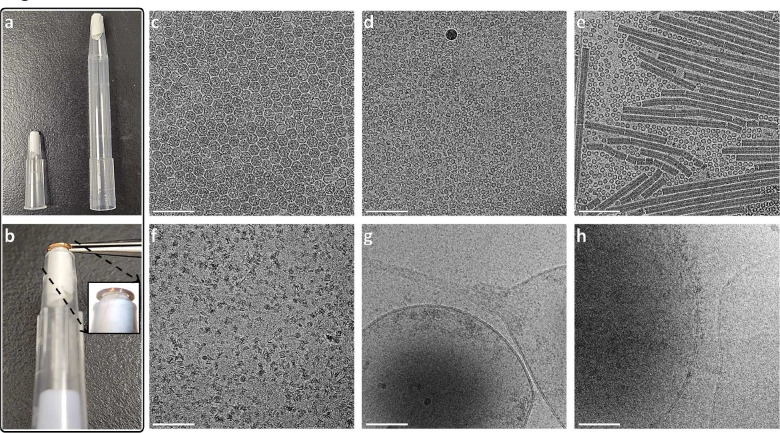
CryoCycle clipped grid blotting with various designs, grids, and samples. **(a)** Two blotting pipette tip sizes: Trimmed 200 μL (left) and 1,000 μL (right) tips, each with a piece of filter paper inserted for blotting 3+ μL of sample. **(b)** A 200 μL blotting pipette tip applied to the same side of the grid as the single particle sample; see orientations in Supplementary Figure 3a. The inset shows the contact between the blotting paper and the grid inside the autogrid ring. *Note*: Blotting for cell samples is from the opposite side of the grid, as shown in Supplementary Figure 3b. **(c-h)** A selection of micrographs of vitreous single particle and cell samples on multiple grid types (gold and carbon) prepared with the CryoCycle method; **(c)** Virus-like Particles (VLPs), **(d)** Apoferritin, **(e)** A protein mixture of apoferritin and tobacco mosaic virus (TMV), **(f)** A p97/selenos complex (conventional preparation comparison in Supplementary Fig. 5), **(g-h)** Thin edges of human retinal pigment epithelial-1 (RPE-1) cells, showing intact membrane bilayers (cryoFLM images shown in Supplementary Fig. 4). Scale bars: 100 nm for (c-h).

**Figure 2 |:**
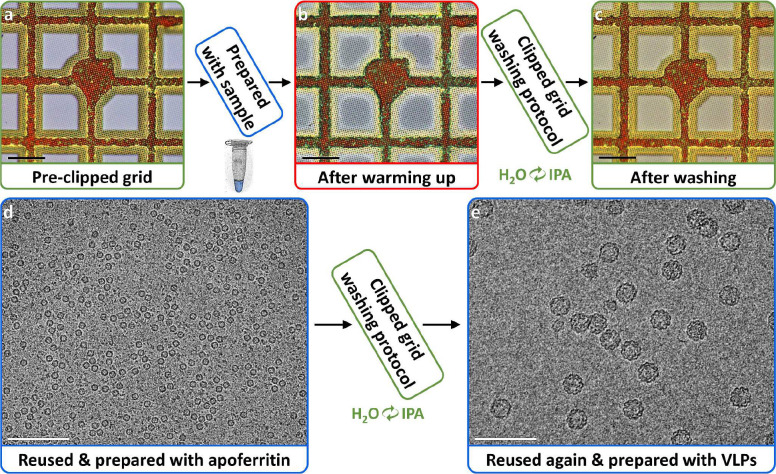
CryoCycle reused clipped carbon grids washing protocol results. **(a)** Squares of a freshly pre-clipped carbon grid. **(b)** Squares of the same grid after vitrifying a sample with the CryoCycle method, warming up, and drying. **(c)** Squares of the same grid after the washing protocol. **(d)** A micrograph of a clipped grid washed and prepared with apoferritin. **(e)** A micrograph from the clipped grid in (d) washed again and prepared with VLPs, showing VLPs of expected sizes. Scale bars: 50 μm for (a-c), 100 nm for (d-e). Supplementary Figure 9 shows CryoCycle reused grid washing for a gold grid. Supplementary Figure 10 shows grid atlases of (d) & (e).

For samples prepared with the CryoCycle blotting method inside a TFS Vitrobot Mark IV chamber (Thermo Fisher Scientific) (Fig. 1c–h, Fig. 2d,e, Supplementary Fig. 4), all time and force parameters were set to zero (Blot Time, Blot Force, Wait Time, and Drain Time) and humidity was set to 80%. The instrument was operated at room temperature. Quantifoil (Quantifoil Micro Tools GmbH) holey carbon, gold, and UltrAuFoil grids were used. Single particle samples were blotted on the same side as sample application following the Procedure in Supplementary Protocol 1 and cell samples were blotted on the opposite side as cell growth (see Supplementary Fig. 3).

For samples prepared conventionally using multiple blotting materials in the TFS Vitrobot Mark IV and Leica EM GP2 chambers (Supplementary Fig. 1), several parameters were varied across different test samples (primarily apoferritin, VLPs, and the protein mixture) including: Quantifoil and C-Flat (Protochips Inc.) carbon and gold grids, blot times from 1 to 4 seconds, and 70–85% humidity.

### CryoEM Grid Screening

Samples were screened on a TFS Glacios cryoTEM (Thermo Fisher Scientific) and a TFS Falcon 3EC camera operating in integrating mode (for most samples) or counting mode using either Smart Leginon Autoscreen (Cheng et al., 2023) or manually in Leginon (Potter et al., 1999). Collection parameters varied across screening sessions; nominal defocus: ~−1.5 to −3 μm, pixelsize: 1.204 Å/pixel, total dose: 50–60 e-/Å^2^.

### CryoEM Processing

The p97/selenos complex prepared with the CryoCycle method (one UltrAuFoil grid and one Quantifoil carbon grid) and screened on a TFS Glacios cryoTEM resulted in 592 micrographs (integrating mode; no frame alignment). Micrographs were processed in Cryosparc v4.4.1 (Punjani et al., 2017) by first CTF correcting, then initial picking with templates and Topaz (Bepler et al., 2019), then iterative 2D classification, manual cleanup, and Topaz retraining, before converging to 24,584 particles after 2D classification (Supplementary Fig. 5c).

The p97/selenos complex prepared conventionally with a TFS Vitrobot Mark IV (one Quantifoil carbon grid) and screened on a TFS Glacios cryoTEM resulted in 2,392 micrographs (counting mode). Micrographs were processed in Cryosparc v4.4.1 (Punjani et al., 2017) before selecting a random subset of 24,584 particles for a final 2D classification (Supplementary Fig. 5b) to match the number of particles from the CryoCycle method dataset.

### SEM imaging

SEM images (Supplementary Fig. 1, 3rd row, 1st column; Supplementary Fig. 2) were collected on an EMCrafts Cube II (EmCrafts Co. Ltd).

### CryoFLM imaging

CryoFLM images (Supplementary Fig. 4) were collected on a Zeiss LSM 900 with Airyscan 2 (Carl Zeiss Microscopy GmbH) configured to excite the sfGFP bound to the CENP-A nucleosomes.

### Verification of Consent

Photo (Supplementary Fig. 12a) and Supplementary Videos 1 & 2 of Viacheslav Serbynovskyi were taken with approval and are shown here with consent.

## Supplementary Material

Supplement 1

## Figures and Tables

**Table 1 |:** CryoCycle savings versus conventional preparation costs. CryoCycle methods save tens to hundreds of thousands of dollars initially and tens of thousands of dollars yearly for a typical cryoEM lab (bold). Added costs are ~$5,000 initially (bold), $0 recurring. Grid savings assume reusing clipped grids 3 times each. Conventional preparation may incur additional losses of unclipped grids, varying with user expertise. Staffing costs or savings may vary depending on how the protocols are implemented.

Potential CryoCycle cost savings					
Item	One-time cost saved	Recurring cost (per item)	Recurring cost (per year)	Recurring cost saved (per year)	Notes
Commercial semi-automated plunge freezer	**~$100,000**	--	--	--	Approx. Leica EM GP or Vitrobot cost. Common in facilities.
Jet freezer	>$500,000	--	--	--	Approx. VitroJet cost. For comparison of an existing method to vitrify clipped grids.
Clipping station	**$2,500**	--	--	--	Approx. cost of a new clipping station.
CryoEM grids	--	$10–20	$6,000	**$4,000**	Assuming 400 grids per year (~8 grids per week) and an avg. cost of $15 per grid.
Clip rings and c-clips	--	$30 per pair	$12,000	**$8,000**	Assuming 400 rings and c-clips per year.
Undipped grid boxes	--	$10	$100	**$100**	Assuming reuse of 10 grid boxes per year.

**Potential additional CryoCycle costs**					
Item	One-time cost	Recurring cost (per item)	Recurring cost (per year)	Recurring cost saved (per year)	Notes
Rotary tool and disk	**$200**	--	--	--	Common in machine shops.
Tweezer sharpening tool	**$100**	--	--	--	Common in cryoEM labs.
CryoCycle tweezers	**$700**	--	--	--	Described in Supplementary Protocol 1
Plunge freezer w/ humidity control	**$4,000**	--	--	--	E.g. Comolli et al., 2012; Depelteau et al., 2020.
