# Supplementary material for: CryoCycle your grids: Plunge vitrifying and reusing clipped grids to advance cryoEM democratization
Source: Res Sq. 2024 Jul 2:rs.3.rs-4415026. Preprint. [Version 1] doi: 10.21203/rs.3.rs-4415026/v1 (PMC11247934; doi:10.21203/rs.3.rs-4415026/v1)
Supplement: Supplement 1 [file NIHPPrs4415026v1-supplement-1.pdf]

SUPPLEMENTARY INFORMATION

Supplementary Figure 1

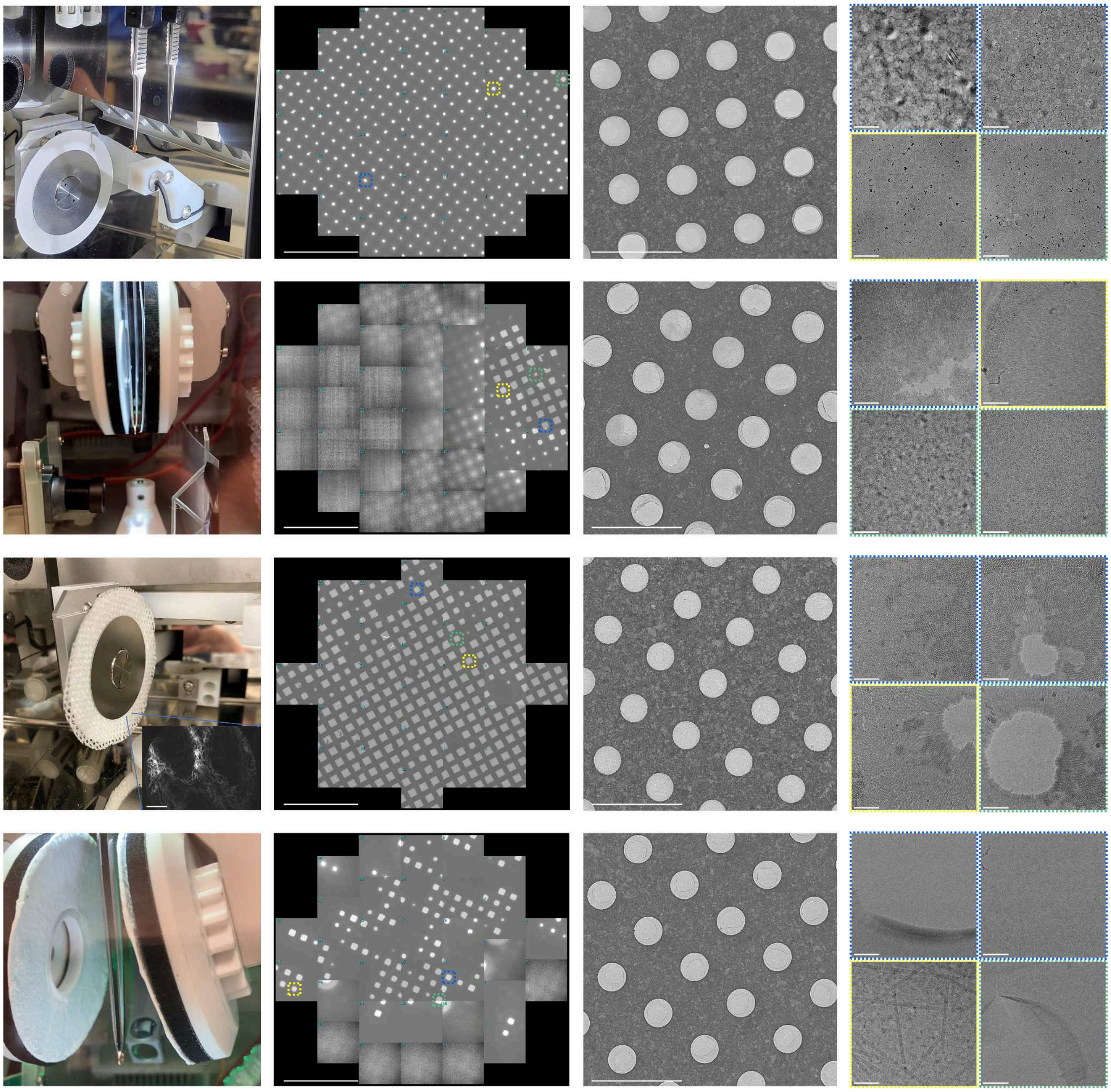

Supplementary Figure 1 | Pre-clipped grid vitrification attempts with conventional plunge freezers and different blotting papers. First column shows blotting papers tested. Second column shows representative grid atlases with high-magnification areas outlined. Third column shows a representative medium-mag image of grid holes. Fourth column shows representative high-mag images of ice in holes. First row shows normal Leica EM GP2 blotting paper. Second row shows normal Vitrobot blotting paper. Third row shows custom holey cotton paper on top of normal Leica EM GP2 blotting paper. Fourth row shows thick cotton blotting paper. Each attempt resulted in predominantly non-vitreous ice and frequent absence of ice at the centers of holes (3rd column). Scale bars: 500 µm, 100 µm, and 100 nm, respectively for 2nd-4th columns; 500 µm for inset SEM image in 3rd row, 1st column.

Supplementary Figure 2

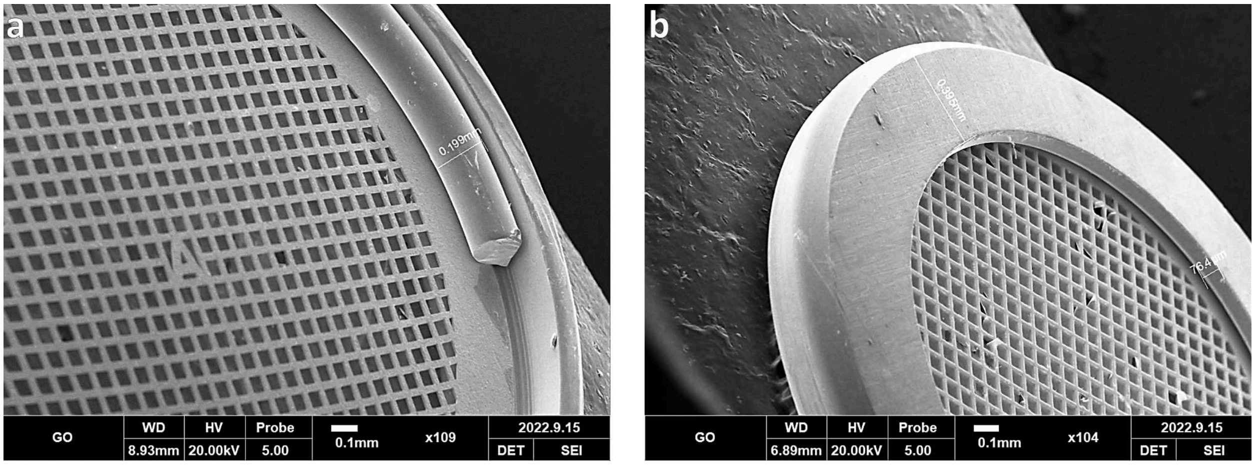

Supplementary Figure 2 | Measurements from angled SEM images of a clipped grid. (a) The front side of the clipped grid shows that the autogrid assembly extends over 100 microns above the grid. **(b)** The back side of the clipped grid shows that the autogrid ring extends many tens of microns above the grid.

Supplementary Figure 3

a) Purified protein samples

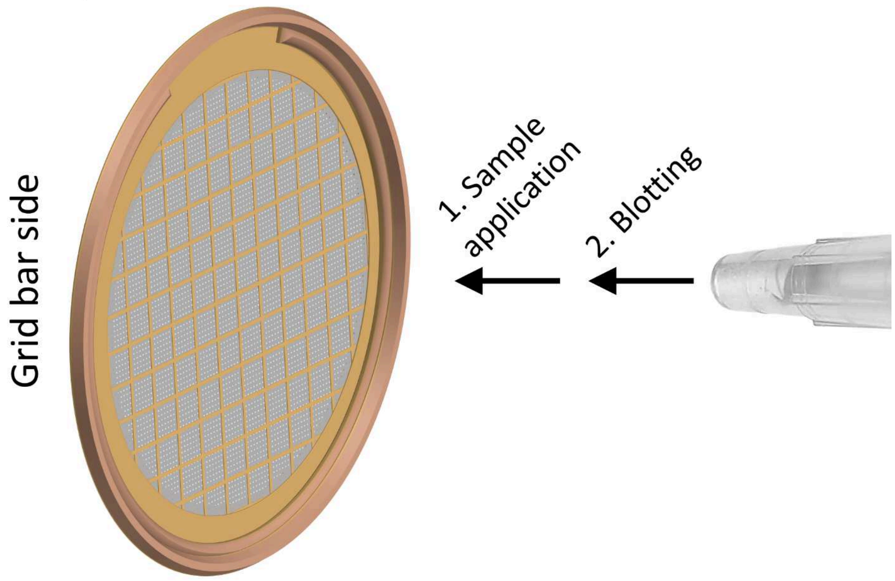

b) Cell samples

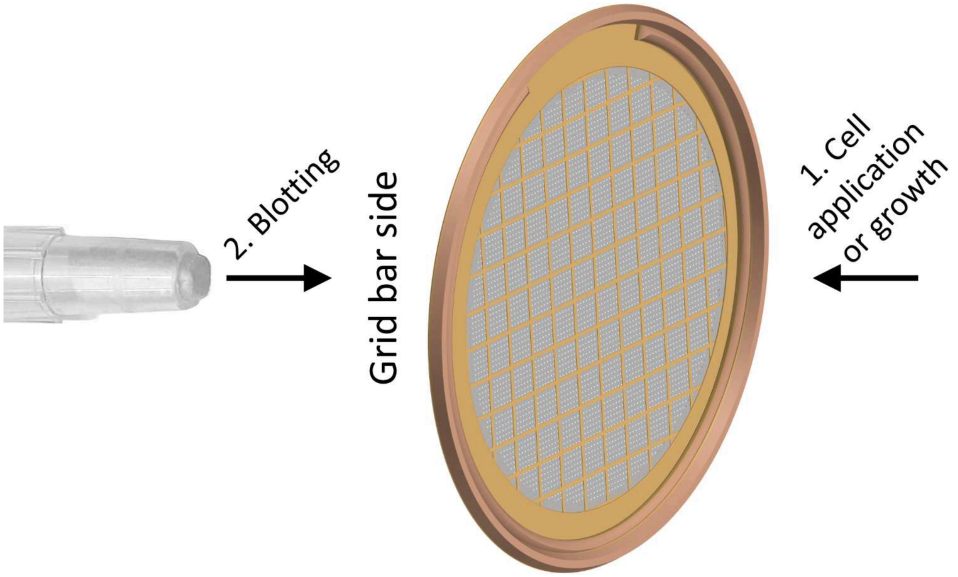

Supplementary Figure 3 | Grid, autogrid ring and c-clip, sample application, and blotting orientation guide. For both purified protein and cell samples, the grid is clipped such that the grid bars are facing the opposite direction as the c-clip. Sample is applied or adherent cells are grown on the non-grid bar side of the grid. The only difference is that purified protein samples are blotted on the same side as sample application **(a)** and cell samples are blotted from the opposite side as cell application/growth **(b)**. Note: The blotting pipette tips and the clipped grids are not to scale.

Supplementary Figure 4

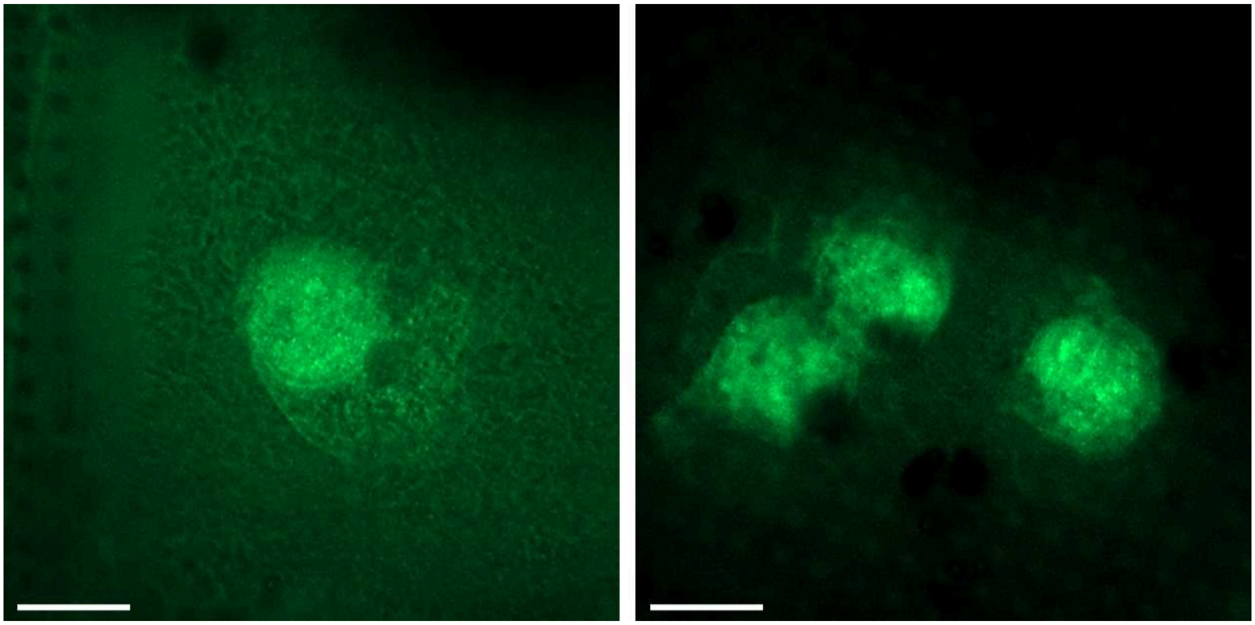

Supplementary Figure 4 | Cryo-fluorescent light microscopy (cryoFLM) images of cells grown on pre-clipped, gold-coated grids. Two cryoFLM images of RPE-1 cells with CENP-A nucleosomes fluorescing in green from the same grid as shown in **Figure 1g,h**. Delineated organelles can be seen. Scale bars are 10 µm.

Supplementary Figure 5

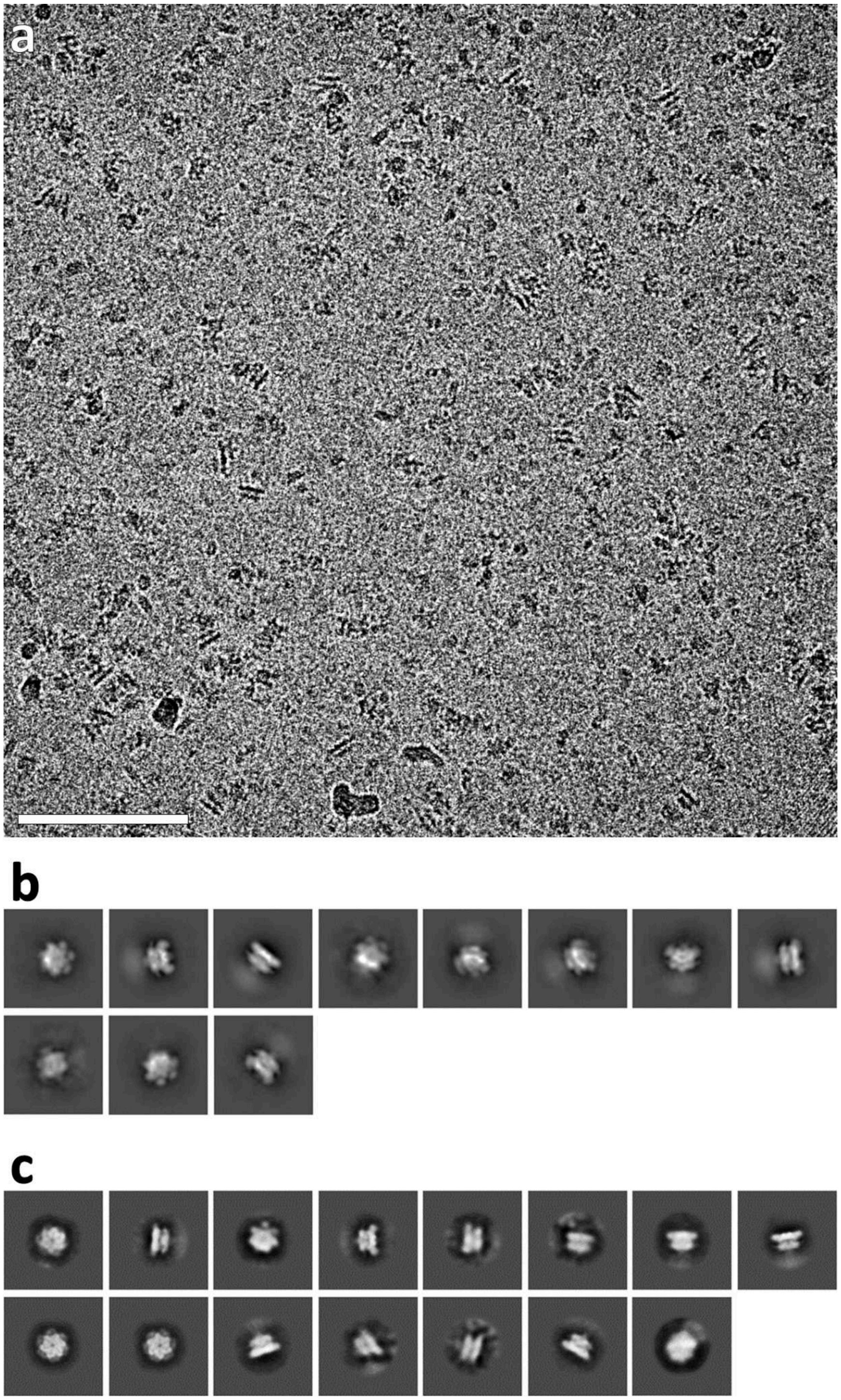

Supplementary Figure 5 | Comparison of conventional plunge freezing of a p97/selenos complex with plunge freezing a preclipped grid with a blotting pipette tip. (a) An example micrograph of the same p97/selenos complex sample in **Figure 1f**, except conventionally prepared with a Vitrobot. **(b)** 2D classes from the dataset in (a). **(c)** 2D classes from the dataset in **Figure 1f**, prepared with a blotting pipette tip. Scale bar: 100 nm.

Supplementary Figure 6

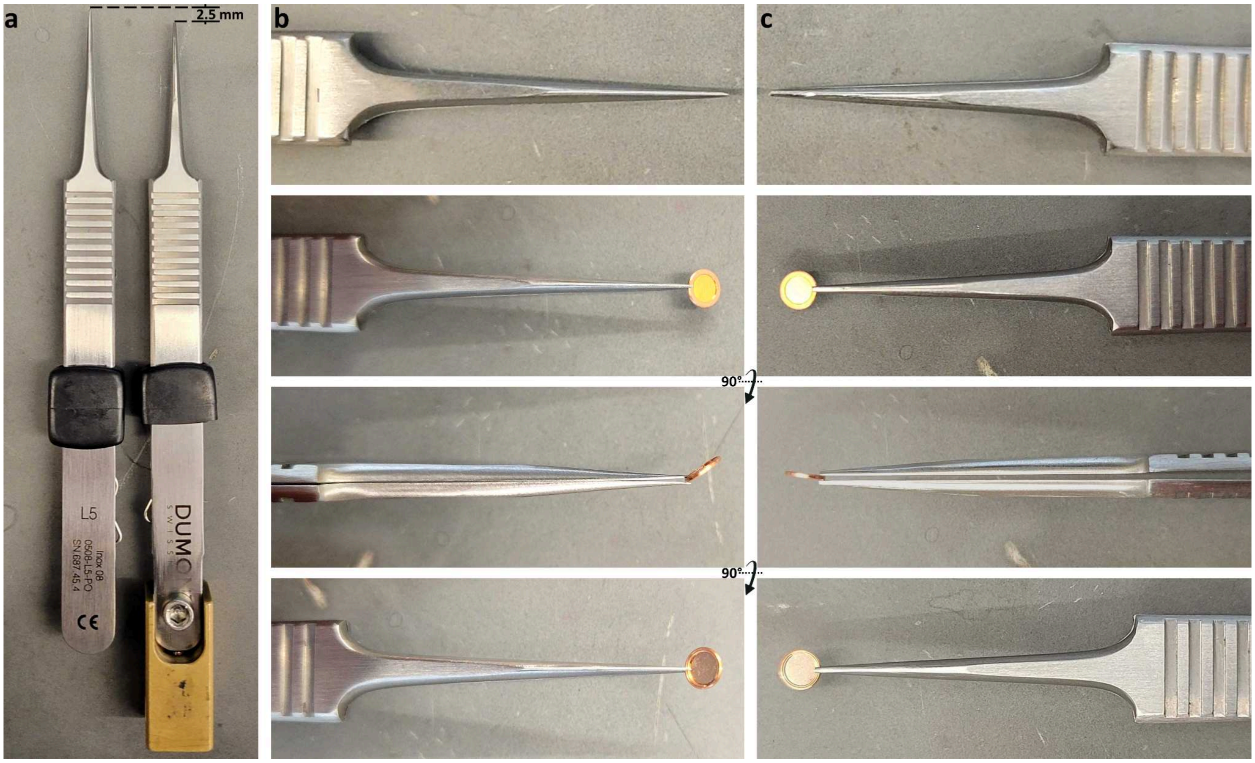

Supplementary Figure 6 | Comparison of unmodified tweezers and modified tweezers for clipped grid freezing. (a) Left: Unmodified Dumont L5 tweezers. Right: Dumont L5 tweezers attached to a Vitrobot mount and modified by trimming off ~2.5 mm of the tip of the tweezer (**Supplementary Protocol 1**). **(b)** Unmodified tweezers; first row shows the fine tip, second row shows the tweezers holding an autogrid from the point of view of the flat side of the ring, third row is rotated 90° from the second row, fourth row is rotated 90° from the third row. **(c)** Modified tweezers; first row shows the trimmed, flattened tip, second row shows the tweezers holding an autogrid from the point of view of the flat side of the ring, third row is rotated 90° from the second row, fourth row is rotated 90° from the third row. In the third row, the unmodified tweezers bend near the tip (b), causing a ~45° rotation of the autogrid which prevents easy blotting and risks the tweezers damaging the grid, while the modified tweezers do not bend and the flattened tip securely holds the autogrid with nearly no rotation (c), allowing for CryoCycle blotting.

Supplementary Figure 7

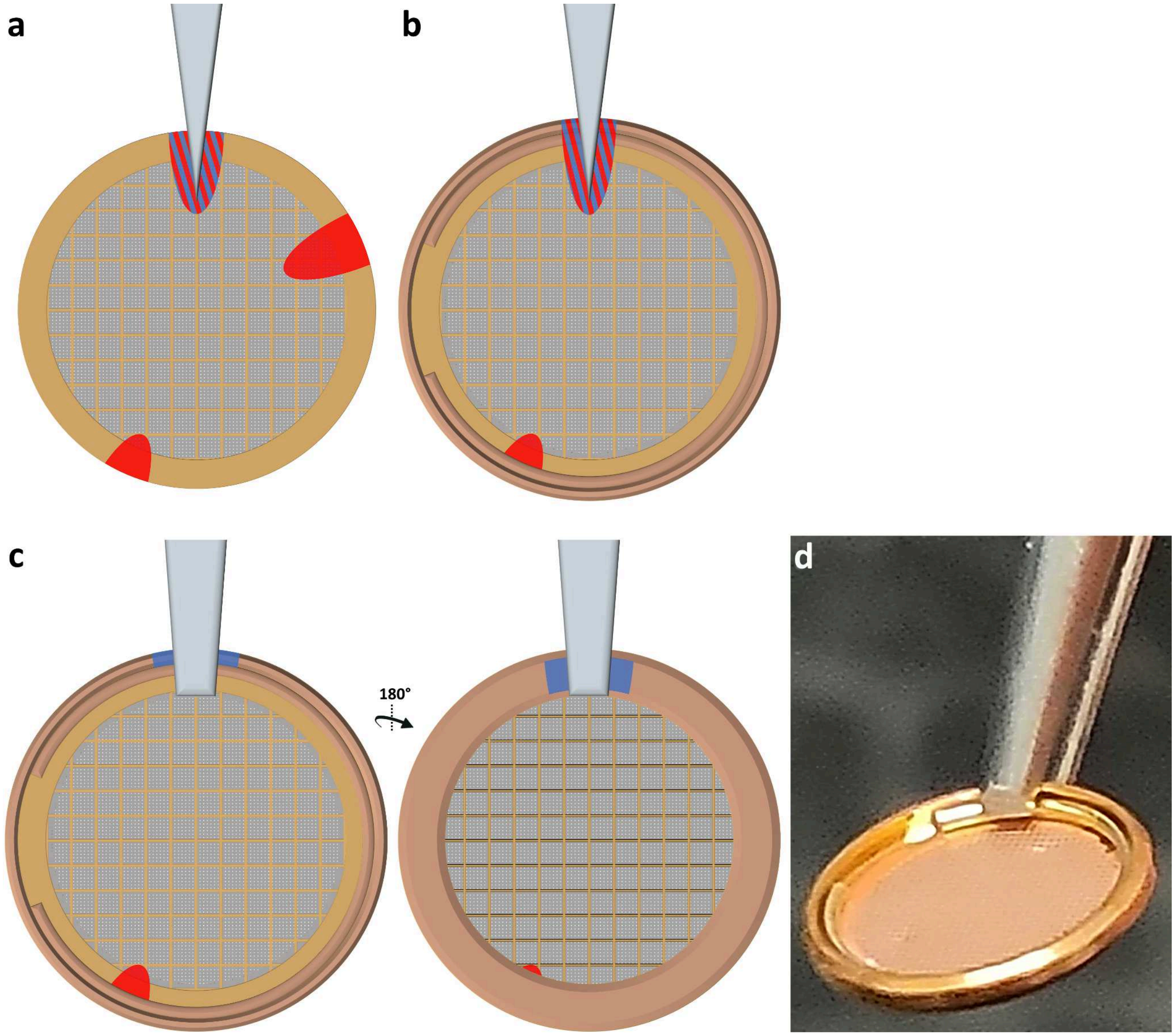

Supplementary Figure 7 | Estimated areas directly affected mechanically and thermally by tweezer handling. (a) A typical grip on an EM grid by fine-tipped tweezers. During several handling steps before imaging (initial grid retrieval, after plasma cleaning/glow discharging to plunge freeze and transfer to a grid box, and handling for clipping), the grid is handled in at least three locations around the rim, potentially causing local physical damage (red). The bottom-left red damage area is from handling at room temperature when it is easier to localize the rim, thus the area is smaller. During freezing, squares near the tweezers are usually not vitrified (red & blue). **(b)** A pre-clipped grid held by fine-tipped tweezers is at high risk of damage near the tweezers, as shown in **Supplementary Figure 6b**, and the grip itself is unstable. **(c)** A clipped grid held by trimmed tweezers (**Supplementary Fig. 6c & Supplementary Protocol 1**) has minimal risk of mechanical damage and minimal risk of vitrification issues (blue) from the tweezers. Both sides of the clipped grid are shown. **(d)** An oblique view of a clipped grid handled by trimmed tweezers as in (c).

Supplementary Figure 8

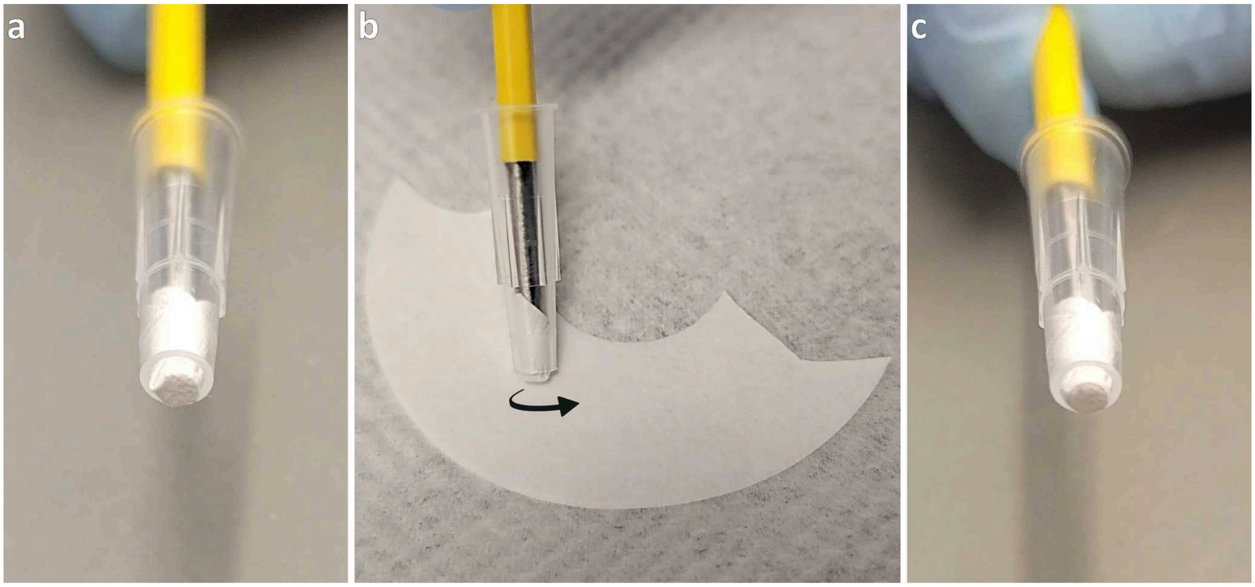

Supplementary Figure 8 | Rounding the edges of blotting paper in a modified pipette tip. During preparation of the blotting pipette tip (**Supplementary Protocol 1**), it is important to ensure that the edges of the blotting paper extruding from the modified pipette tip are rounded and smoothed to facilitate blotting inside of the clip ring (**Fig. 1b**). To do this, position the assembly at ~60° with respect to a flat surface with the metal rod inserted and roll the end of the blotting paper on clean filter paper until smooth. **(a)** 200 µL blotting pipette tip before rolling. **(b)** Rolling the blotting tip at a 60° angle on a flat, clean surface. **(c)** Blotting pipette tip after rolling at 60° and pressing at 90°.

Supplementary Figure 9

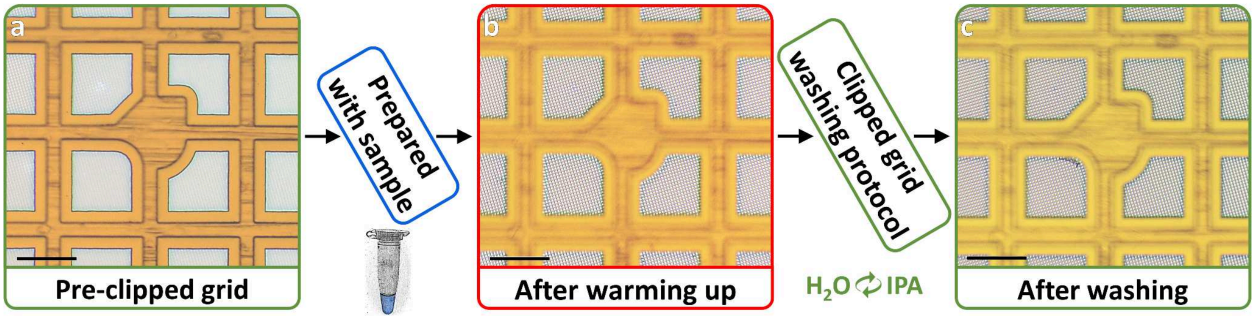

Supplementary Figure 9 | CryoCycle reused clipped gold grids washing protocol results. (a) Squares of a freshly pre-clipped gold grid. **(b)** Squares of the same grid after vitrifying a sample with the CryoCycle method, warming up, and drying. **(c)** Squares of the same grid after the washing protocol.

Supplementary Figure 10

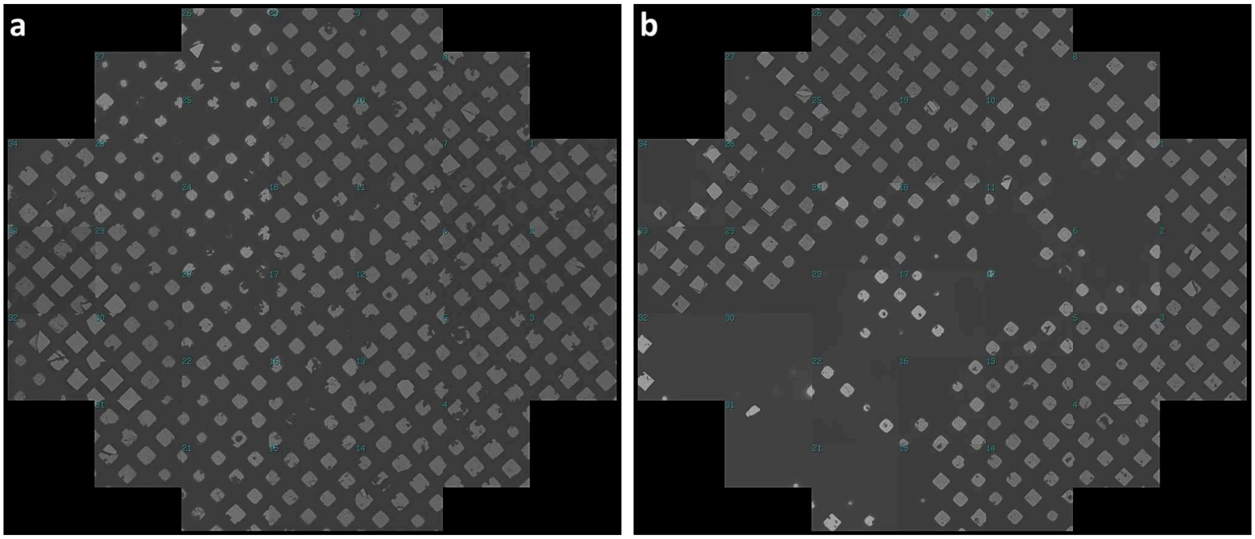

Supplementary Figure 10 | Grid atlases of a carbon grid after washings. (a) Grid atlas of the carbon grid with apoferritin shown in **Figure 2d** that has already been washed and reused once - after preparation with VLPs - showing that the vast majority of grid squares are intact. **(b)** Grid atlas of the same grid shown in **Figure 2e** that was washed and reused again and prepared with VLPs showing a similar small number of broken grid squares as in (a). The previous sample was not found to be on the grid after the washings.

Supplementary Figure 11

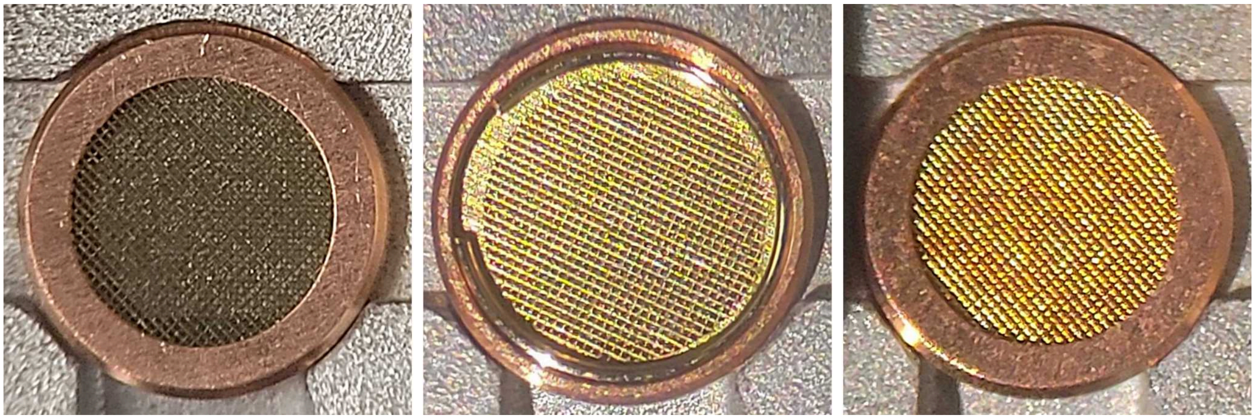

Supplementary Figure 11 | Examples of grids clipped at room temperature. Photos of one carbon (left) and two gold grids (middle, right) clipped at room temperature. The grids show virtually no damage due to minimal handling.

Supplementary Figure 12

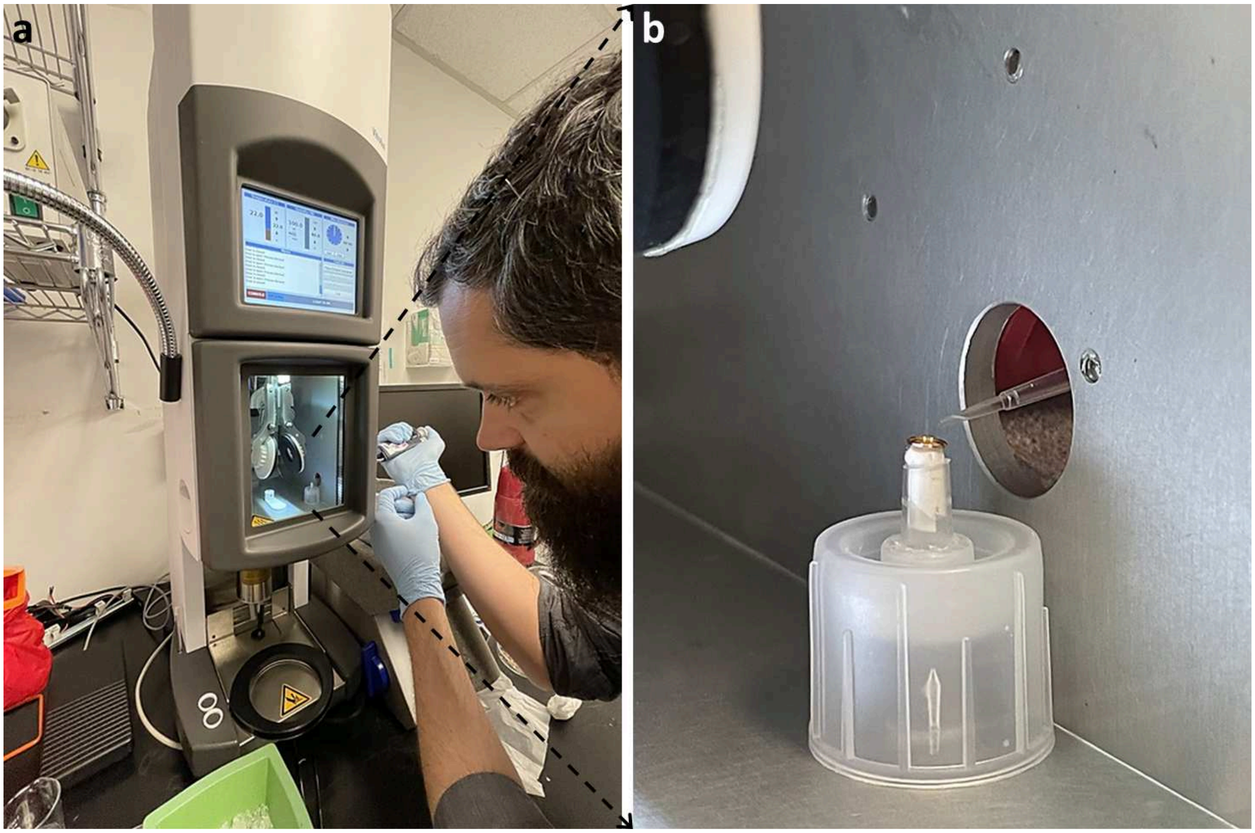

Supplementary Figure 12 | CryoCycle-gravity development. Ongoing development of a CryoCycle setup that attempts to use gravitational force in place of manual blot force to make the method more universally reproducible. **(a)** Photo of Viacheslav Serbynovskyi testing a CryoCycle-gravity setup in a TFS Vitrobot where the chamber is only being used for humidity control. Sample is applied on top of a stabilized blotting pipette tip for through-grid wicking. Seconds after application and wicking, the grid is plunged by hand into LN2-cooled liquid ethane outside of the Vitrobot (out of view in the photo). **(b)** Zoom-in of the modified pipette tip setup, which is also visible at the bottom of the custom Allen wrench storage base shown in **Supplementary Figure 13, item #19**.

Supplementary Figure 13

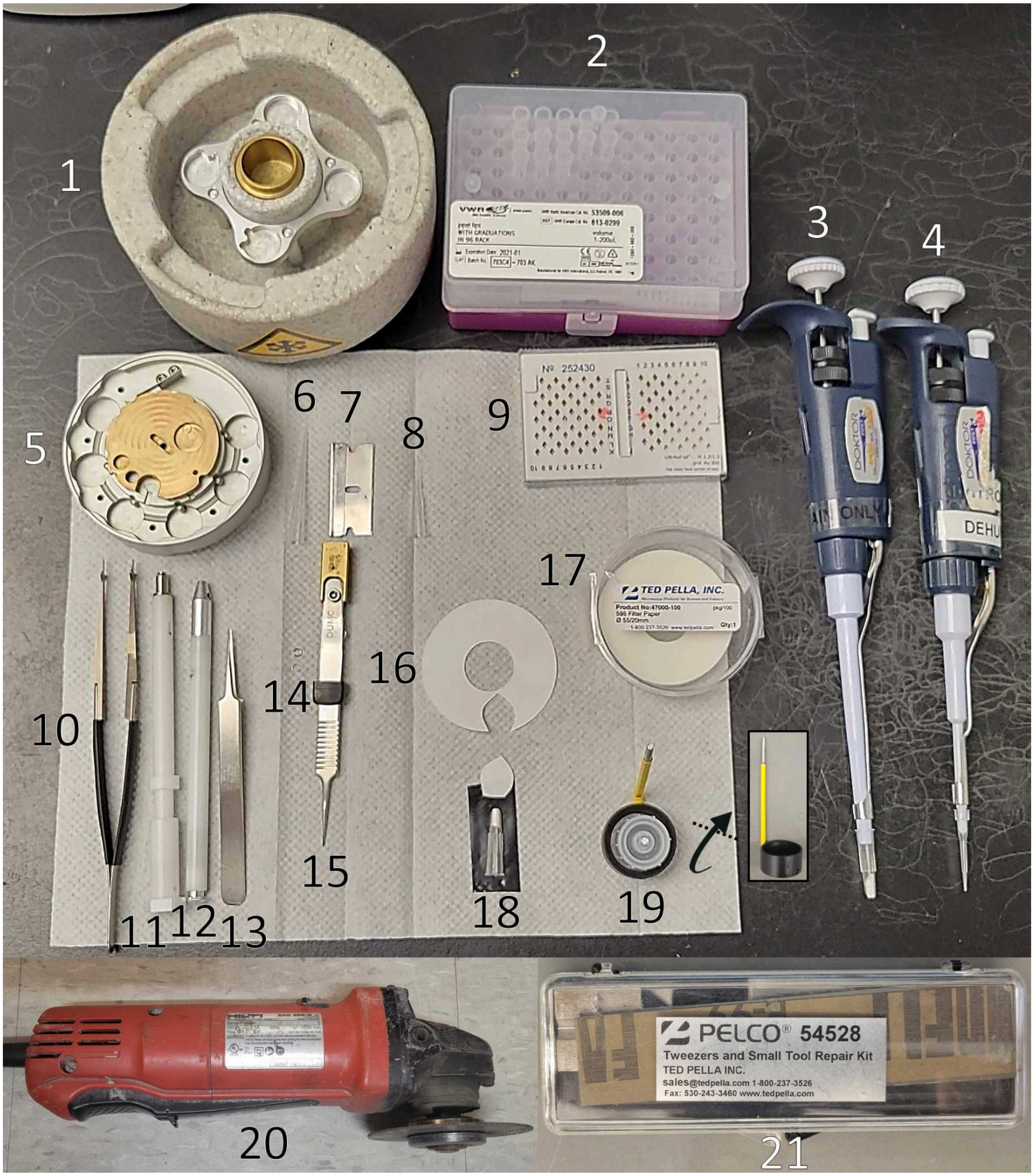

Supplementary Figure 13 | Items recommended for preparing for CryoCycle blotting along with numbers corresponding to Supplementary Protocol 1. *Note*: CryoCycle modified pipette tips may be created with any pipette tip that has an inner diameter smaller than 3 mm; A 200 µL pipette tip is shown here. 1,000 µL and 200 µL pipette tips are shown in **Figure 1a,b**. *Note*: an autogrid clipping station (item #5) is not required; Room temperature grids can be clipped on a flat surface without a clipping station.

Supplementary Figure 14

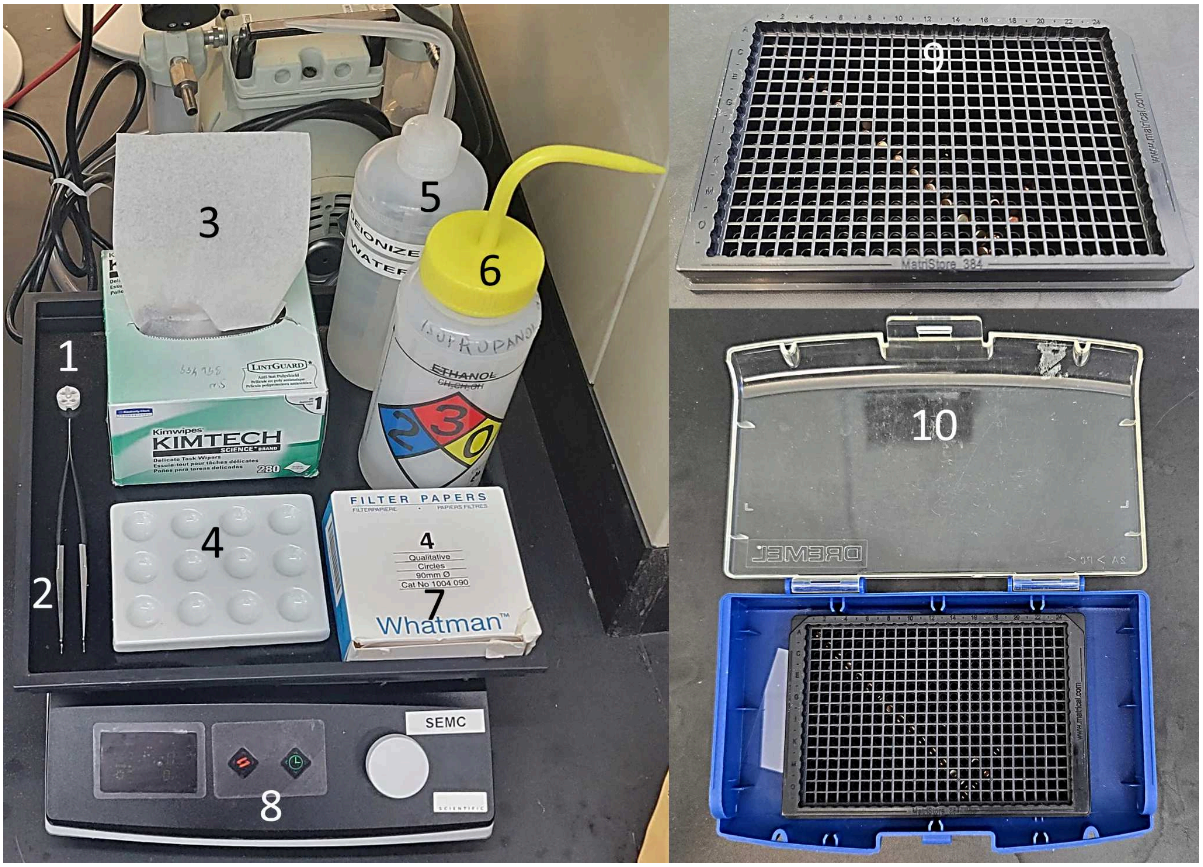

Supplementary Figure 14 | Items recommended for the CryoCycle clipped grid washing protocol along with numbers corresponding to Supplementary Protocol 2.

Supplementary Figure 15

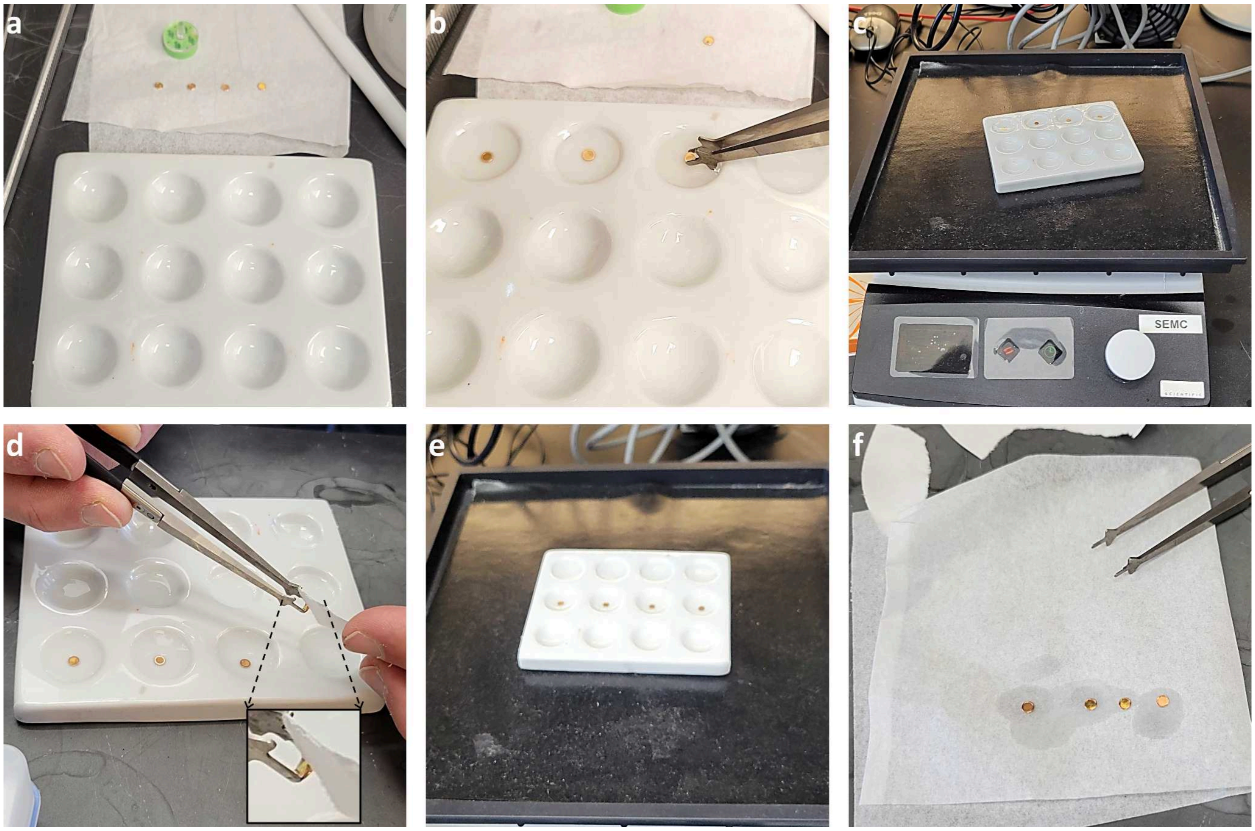

Supplementary Figure 15 | Clipped grid washing CryoCycle protocol for reusing grids, as described in Supplementary Protocol 2. Shown here are four used clipped grids that are first warmed up **(a)**, then submerged and washed in water while shaking for 5 minutes **(b-c)**, then blotted on the side **(d)**, then submerged and washed in isopropanol while shaking for 5 minutes **(e)**, then submerged and washed in isopropanol for a second time while shaking for 5 minutes (not shown), then dried on a Kimwipe tissue **(f)**.

Supplementary Figure 16

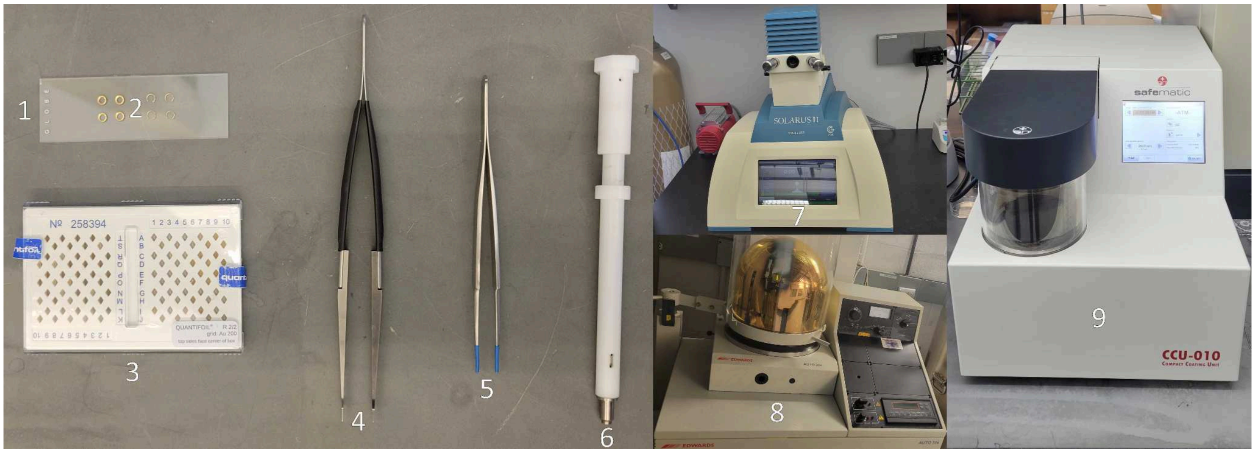

Supplementary Figure 16 | Items recommended for preparing cells on clipped grids for use with CryoCycle cell preparation along with numbers corresponding to Supplementary Protocol 3. *Note*: The grid handling tweezers with soft tip coating (item #5) were prepared by molding heat-shrink tubing onto the tips using a flame; this addition prevents the gold-coated c-clips from being scratched by the tweezers upon insertion into the c-clip insertion tool (item #6).

Supplementary Video 1

https://nysbc-my.sharepoint.com/:v:/g/personal/anoble_nysbc_org/Ecx-2ramF6FAkK9W5I0Vjk4BRcCW8ZXhQq_hBjEpSET9Zw?nav=eyJyZWZlcnJhbEluZm8iOnsicmVmZXJyYWxBcHAiOiJPbmVEcml2ZUZvckJ1c2luZXNzIiwicmVmZXJyYWxBcHBQbGF0Zm9ybSI6IldlYiIsInJlZmVycmFsTW9kZSI6InZpZXciLCJyZWZlcnJhbFZpZXciOiJNeUZpbGVzTGlua0NvcHkifX0&e=AXqRei

Supplementary Video 1 | Video of Viacheslav Serbynovskyi using the CryoCycle blotting method to vitrify a single particle sample using a 1,000 µL blotting pipette tip. (0:00) Prepare trimmed tweezers gripping a clipped grid and mounted into a plunge freezing device, LN2-cooled liquid ethane, a blotting pipette tip. **(0:19)** Pipette 3 µL of sample onto the center of the c-clip side of the clipped grid. **(0:50)** Blot the clipped grid just inside of the autogrid assembly using a blotting pipette so that the extruded filter paper makes complete contact with the face of the grid. **(1:07)** Plunge the clipped grid into liquid ethane. **(1:26)** Detach the tweezers from the plunging device, transfer the grid to the LN2, and place the grid into a clipped grid box.

Supplementary Video 2

https://nysbc-my.sharepoint.com/:v:/g/personal/anoble_nysbc_org/ER8ZXaFLYnVAj1no8NHZ7pUB-ZW-rY2EEi7qQdLjz-x78w?nav=eyJyZWZlcnJhbEluZm8iOnsicmVmZXJyYWxBcHAiOiJPbmVEcml2ZUZvckJ1c2luZXNzIiwicmVmZXJyYWxBcHBQbGF0Zm9ybSI6IldlYiIsInJlZmVycmFsTW9kZSI6InZpZXciLCJyZWZlcnJhbFZpZXciOiJNeUZpbGVzTGlua0NvcHkifX0&e=bgAC5N

Supplementary Video 2 | Video of Viacheslav Serbynovskyi assembling a 200 µL blotting pipette tip. **(0:00)** Trim the end of a pipette tip. **(0:20)** Cut a ~1 cm circle out from the filter paper, **(0:30)** mold it to the end of the metal rod, and **(0:38)** insert it slowly and without twisting into the large end of the trimmed pipette tip until it extends 1 mm beyond the small end. **(0:49)** Tilt the assembly about 60° to the surface of a flat filter paper and roll the edge of the extruded filter paper until it is well-rounded. **(0:54)** With the assembly tilted 90° to the surface, firmly press down. **(0:59)** Twist the rod about one eighth of a turn in one direction, then twist in the other direction while pulling the rod out of the pipette tip.

Supplementary Protocol 1: Vitrifying Clipped Grids

This protocol is for preparing clipped grids (blotting and vitrifying) with single particle-like samples.

Reagents and Materials (Supplementary Figure 13)

1. Vitrification dewar (e.g. Thermo Fisher Scientific, catalog number: FEI0815NB)
2. Pipette tips (e.g. VWR, catalog number: 53509-006)
3. Pipette for blotting (e.g. Rainin Classic PR-200, catalog number: 17008652)
4. Pipette for sample application (e.g. Rainin Classic PR-10, catalog number: 17008649)
5. (Optional) AutoGrid assembly workstation clipping hub (e.g. Thermo Scientific, catalog number: 1000068)
6. An example pipette tip
7. Razor (e.g. EMS, catalog number: 71960)
8. An example trimmed pipette tip
9. CryoEM grids (e.g. Quantifoil UltrAuFoil R 1.2/1.3 Au 300 mesh grids, catalog number: N1-A14nAu30-01)
10. (Optional) AutoGrid tweezers (e.g. Thermo Scientific, catalog number: 9432 909 97631)
11. C-clip insertion tool (e.g. Thermo Scientific, catalog number: 9432 909 97571)
12. Grid container tool (e.g. Thermo Scientific, catalog number: 9432 909 97671)
13. Grid handling tweezers (e.g. Dumont, catalog number: 0208-5-PO)
14. Autogrid ring and c-clip (e.g. Thermo Scientific, catalog numbers: 1036173 and 1036171)
15. Modified grid plunging tweezers (e.g. Ted Pella, catalog number: 47000-500)
16. An example filter paper with a cutout removed (e.g. Ted Pella 595 filter paper, catalog number: 47000-100)
17. Filter paper (e.g. Ted Pella 595 filter paper, catalog number: 47000-100)
18. An example assembled trimmed pipette tip with filter paper. An additional filter paper cutout is placed above the pipette tip.
19. A cylindrical metal rod (~2 mm diameter) used for inserting the filter paper cutout into the modified pipette tip (shown in **Supplementary Figure 13** attached to a custom storage base made from an LN2 tank cap and a modified lab squeeze bottle; A modified Allen wrench that was rounded by a rotary tool is shown in the top-down view and inset side view).
20. Rotary tool to prepare tweezers, if necessary (e.g. Hilti angle grinder, catalog number: DAG 450-D)
21. Tweezer sharpening tool to prepare tweezers, if necessary (e.g. Pelco Tweezers and Small Tool Repair Kit, catalog number: 54528)
22. Several additional reagents and materials not shown in **Supplementary Figure 13** are required, including: A plunge freezing device with humidity-controlled chamber, a glow discharger, liquid nitrogen (LN2), ethane gas (99.9+% purity), cryo dewars, cryoEM sample, clipped grid boxes, clock for measuring blotting time, scissors, lab gloves, and eye protection.

Preparation of tweezers

1. Before beginning, check if your tweezers require trimming by gripping a clipped grid on the rim as in **Supplementary Figure 6**. If the tips of the tweezers flex causing the clipped grid to rotate as in **Supplementary Figure 6b, third row**, then the tweezers require trimming. If the tips of the tweezers do not flex and the clipped grid does not rotate significantly as in **Supplementary Figure 6c, third row**, then the tweezers do not require trimming. If the tweezers require trimming, continue with the following steps.
2. Mark 2.5 mm from the tweezer tip with a permanent marker.
3. Using all proper safety measures, position the tips of the tweezers 90° to the plane of the spinning rotary tool disk. Carefully move the tweezer tips into the rotary tool disk until the tips are shaved down to the marker line.
4. Sharpen the tweezers with a sharpening tool. Ensure that the tips of the tweezers that will grip the autogrid remain square, as shown in **Supplementary Figure 7c,d**. The modified tweezers should grip a clipped grid as shown in **Supplementary Figure 6c, third row**.

Preparation of blotting pipette tip (Supplementary Video 2)

1. Put on lab gloves. Clean all handling tools for grids and filter paper before use.
2. Trim the end of a pipette tip with a razor so that the inner diameter of the opening is 3 mm. Ensure that the end of the pipette tip is smooth.
3. Cut a ~1 cm circle out from the filter paper.
4. Mold the filter paper cutout to the end of the rod.
5. With the filter paper cutout molded to the end of the rod, insert the assembly into the large end of the trimmed pipette tip until the filter paper extends 1 mm beyond the small end. Insert the assembly slowly and without twisting so as to not tear the filter paper.
6. Place a piece of filter paper on a flat surface. Tilt the rod, filter paper, and pipette tip assembly about 60° to the surface and roll the edge of the extruded filter paper on the flat filter paper while pushing on the rod so that the extruded filter paper is well-rounded (**Supplementary Fig. 8; Supplementary Video 2**). Tilt the assembly to be perpendicular to the flat surface and firmly press down on the rod so that the extruded filter paper is flat on the end.
7. To remove the rod, twist the rod about one eighth of a turn in one direction, then twist in the other direction while pulling the rod out of the pipette tip.
8. Store the blotting pipette tip in a clean, dry environment until ready for use.
   Note: Prepare one blotting pipette tip per grid. Do not reuse blotting pipette tips.

Procedure for blotting and vitrifying clipped grids (Supplementary Video 1)

1. Prepare the following: trimmed tweezers, freshly glow-discharged clipped grids (grid bar side opposite to the c-clip side as shown in **Supplementary Figs. 2, 3, & 6c**), one blotting tip per clipped grid, sample for application (3 µL per grid), LN2-cooled liquid ethane in the vitrification dewar, a plunge freezing device (disable blotting, set humidity

to 80+%), and a clock for measuring blotting time (a clock with audible ticks every second is recommended).

2. Grip a clipped grid with the trimmed tweezers and mount the tweezers onto the plunging device.
3. Pipette 3 μL of sample onto the center of the c-clip side of the clipped grid.
4. Use a blotting pipette tip to blot the grid inside of the autogrid ring and c-clip on the same side that the sample was applied (c-clip side) for a desired amount of time (typically 1 - 4 seconds) (**Fig. 1b**).
   Note: Ensure that the flat blotting paper fully contacts the flat grid. Ideally, the blotting pipette tip should be oriented exactly 90° relative to the plane of the grid.
   Note: Blot force is determined by the pressure on the grid exerted by the user's hand.
   Note: Avoid contacting the blotting paper with any object other than the grid when inserting into the preparation chamber so as to not deform the flat, rounded blotting paper.
5. Plunge the clipped grid into liquid ethane.
6. Detach the tweezers from the plunging device, transfer the grid to the LN2, and place the grid into a clipped grid box.
   Note: When transferring the clipped grid from the liquid ethane to the LN2, orient the face of the clipped grid to be perpendicular to the direction of motion so as to not transfer ethane along with the clipped grid, as shown in **Supplementary Video 1**.

Supplementary Protocol 2: Washing Clipped Grids

This protocol is for cleaning clipped grids for reuse with different single particle samples without clipped grid disassembly.
Note: Ensure that the structural integrity of each autogrid ring and c-clip has not been impaired between the retrieval from an autoloading microscope and insertion into the subsequent autoloading microscope so as to not damage the microscopes.
Note: This protocol has not been tested with cell samples.

Reagents and Materials (Supplementary Figure 14)

1. Used clipped grids
2. Tweezers for handling autogrids (e.g. Thermo Scientific, catalog number: 9432 909 97631)
3. Low-lint tissues (e.g., Kimtech, catalog number: 34155)
4. Porcelain spot plate (e.g. Science Lab Supplies, catalog number: 3765-2)
5. Highly purified water (e.g. from a Hydro Picotap, catalog number: JKLCN0202N2H-FC)
6. Isopropanol 99+% (e.g. Sigma Aldrich, catalog number: PX1830-4)
7. Filter papers (e.g., Whatman, catalog number: 1004090)
8. Rotator/Shaker (e.g. Thermo Scientific, catalog number: 88880025)
9. (Optional) Reused grid organization tray with numbers and letters (e.g. Fluotics Matrical Matristore Microtube Rack, catalog number: MatriStore 384 Tube Rack)
10. (Optional) Storage box for the reused grid organization tray (e.g. Dremel, catalog number: 2610923299)

Procedure for cleaning clipped grids for reuse

1. Place the used clipped grids on a tissue and let them warm up to room temperature (**Supplementary Fig. 15a**).
2. Fill a row of wells in the porcelain spot plate with purified water; one for each grid to be washed.
3. Grip each clipped grid, one by one, by the outer rim of the clip ring. Submerge each clipped grid under water in their separate wells, as shown in **Supplementary Figure 15b**.
4. Place the spot plate on the rotator and rotate at 60 rpm for 5 minutes (**Supplementary Fig. 15c**).
5. Fill the next row of wells in the spot plate with isopropanol; one for each grid being washed.
6. Grip each submerged clipped grid, one by one, by the outer rim of the clip ring. Remove each grid from the water and blot with filter paper on the side of the clip ring (i.e. not touching the grid) as shown in **Supplementary Figure 15d**. Submerge each clipped grid under isopropanol into separate wells.
7. Place the spot plate on the rotator and rotate at 60 rpm for 5 minutes (**Supplementary Fig. 15e**).

8. Repeat steps 5 through 7.
9. Place grids on a tissue to dry (**Supplementary Fig. 15f**).
10. Proceed with the Procedure in **Supplementary Protocol 1** or store the grids in an organization tray and box (**Supplementary Figure 14, items #9 & 10**) for future use.

Recommendations

We recommend that new CryoCycle users verify the single particle CryoCycle protocol (**Supplementary Protocols 1 & 2**) in their hands as follows:

1. Prepare four new pre-clipped cryoEM grids with a standard sample, such as apoferritin (**Supplementary Protocol 1**).
2. Wash the grids for reuse (**Supplementary Protocol 2**).
3. Apply buffer as the next sample to two of the reused grids as a negative control to ensure no protein remains and apply a second standard sample, such as aldolase, as a positive control to the other two reused grids to ensure that only the second sample remains (**Supplementary Protocol 1**).
4. (Optional) Test how many times the grids can be reused (repeat steps 2 & 3).

We recommend keeping track of the following when reusing clipped grids:

- Grid type (brand, substrate, mesh size, hole size)
- Number of times reused
- Previous sample(s)
- Previous instruments & procedures used (washing protocol, plasma cleaning/glow discharge, freezing protocol, microscopes)
- Previous person(s) who handled the grid

We recommend separating clipped grids for reuse immediately after unloading from the microscope and storing them in an organization tray and box (e.g. **Supplementary Figure 14, items #9 & 10**). Use the organization tray numbering system to record the reused grid details. Washed grids can be stored in a separate organization tray.

We recommend reusing clipped grids for screening visually distinct samples to avoid misinterpretation if washing was incomplete, and only with samples that do not interact with one another.

Supplementary Protocol 3: Preparing Pre-clipped Grids for Cells

This protocol is for preparing cell-compatible pre-clipped grids. The goal of this protocol is to gold-coat autogrid rings and c-clips so that cell samples can be grown on or applied to the pre-clipped grids without cytotoxicity from the autogrid assembly. We initially found that multiple coats of gold are required to coat autogrid rings and c-clips before clipped grid assembly, and so developed a less costly version of the protocol with an initial coating of carbon and final coating of gold.

Reagents and Materials (Supplementary Figure 16)

1. Glass slide (e.g. EMS, catalog number: 71883-01)
2. Autogrid ring and c-clip (e.g. Thermo Scientific, catalog numbers: 1036173 and 1036171)
3. CryoEM grids (e.g. Quantifoil UltrAuFoil R 1.2/1.3 Au 300 mesh grids, catalog number: N1-A14nAu30-01)
4. (Optional) AutoGrid tweezers (e.g. Thermo Scientific, catalog number: 9432 909 97631)
5. Grid handling tweezers with soft tip coating (e.g. Dumont, catalog number: 0208-5-PO)
6. C-clip insertion tool (e.g. Thermo Scientific, catalog number: 9432 909 97571)
7. Plasma cleaner (e.g. Gatan Inc., Gatan Solarus II Model 955)
8. Gold evaporator (e.g. Edwards, Auto 306 Vacuum Coater operated at 3 kV, 50 mA)
9. Carbon evaporator (e.g. Safematic, catalog number: CCU-010)

Procedure for preparing cell-compatible pre-clipped grids

1. Plasma clean both sides of the autogrid rings, autogrid c-clips, and grids (oxygen and argon for 7 seconds).
2. Place the autogrid rings and c-clips in the carbon evaporator and coat them with ~20 nm of carbon on both sides.
3. Place the autogrid rings and c-clips in the gold evaporator and coat them with several nanometers of gold on both sides.
4. Clip grids using the carbon+gold coated autogrid rings and c-clips at room temperature. Use the soft tip coated tweezers for inserting the c-clip into the c-clip insertion tool.
5. Plasma clean both sides of the pre-clipped grids (oxygen and argon for 7 seconds).
6. Grow or apply cells to the grid bar side of the grid (opposite to the c-clip side as shown in **Supplementary Fig. 3**).
7. Proceed with the Procedure in **Supplementary Protocol 1**, except skip step 3 and blot from the side opposite to sample application as shown in **Supplementary Figure 3**.
